# Cruelty to Babies

**Published:** 1874-11

**Authors:** 


### CRUELTY TO BABIES.

There are several forms in civilized society. The one to which we now refer is practiced daily by mothers and nurses in exposing the eyes of their children to the dazzling light of the sun. The child riding in its little carriage is often made to face the sun, unshaded and unprotected, just as long as suits the convenience of its heedless attendant. The little one must suffer a degree of discomfort and pain which its mother could not bear a moment.

If a very little baby, and carried in the nurse's arms, it is made to lie on its back, its face upturned, the direct rays of the sun piercing its eyes. Through the instinct of self-preservation the baby-hands would come to the protection of the baby-eyes; but they are bound down by cloaks and blankets, and the helpless one must suffer until the nurse happens to change its position.

None can tell the amount of injury done to the delicate organ of sight by such thoughtless cruelty.

